# Cæsarean Section in Great Britain and Ireland

**Published:** 1913-06

**Authors:** 


**Cæsarean Section in Great Britain and Ireland.** By AMAND ROUTH, M.D., F.R.C.P. Pp. 233. London: Sherratt and Hughes. 1911. 5s. net.

Dr. Routh's work contains tables of 1,282 cases of Cæsarean section by over one hundred living obstetricians and gynæcologists in Great Britain and Ireland, and is one of more than ordinary usefulness. It presents the views of obstetricians of all countries and schools on the indications and technique of this operation, and in the summaries at the end of the sections gives the general consensus of opinion on the more doubtful points.

The various indications for the operation are considered in detail. Naturally, the one which takes up most space is obstructed labour from contracted pelvis. The place of Cæsarean section in the treatment of this condition is most fully discussed. Comparison is made with the various alternative methods of treatment—induction of labour, embryotomy, pubiotomy; the difficult question of infected and "suspect" cases is throughly gone into, and the relative merits of the different methods of operating clearly set forth. The ordinary transperitoneal operation is the one most generally approved in this country; infected cases which need Cæsarean section, where an extra-peritoneal method would be most desirable, being best dealt with by Cæsarean section followed by hysterectomy.

The prevailing opinion on sterilisation as an accompaniment of Cæsarean section appears to be that the decision as to its adoption should be left, after the position has been clearly explained, to the patient, the favourite method of effecting it being by removal of the Fallopian tubes in whole or in part. A useful summary of conclusions brings this part of the subject to a close.

In the following sections the circumstances in which Cæsarean section may be called for in cases of uterine or pelvic tumours, uterine hemorrhage or constitutional crisis are considered, and an account is given of the history and present position of the operation of vaginal Cæsarean section.

The paper concludes with a series of mortality tables, showing the gradual diminution in the death-rate during the last decade, its variation according to the condition for which the operation is done and the type of procedure adopted, and the enormous influence on it of previous infection of the genital tract.

Appended is a very careful summary of the 1282 cases on which these observations are founded. The work is a complete account of the operation of Cæsarean section, and there is no point in connection with it on which ample material for forming an opinion and assistance thereto may not be obtained.

